# Low bleeding acceptance is associated with increased death risk in patients with atrial fibrillation on oral anticoagulation

**DOI:** 10.1007/s11239-023-02878-8

**Published:** 2023-08-19

**Authors:** Gabriela Rusin, Małgorzata Konieczyńska, Joanna Natorska, Krzysztof Piotr Malinowski, Anetta Undas

**Affiliations:** 1 Department of Neurology, University Hospital, Kraków, Poland; 2 Department of Diagnostic Medicine, John Paul II Hospital, Kraków, Poland; 3 Department of Thromboembolic Disorders, Institute of Cardiology, Jagiellonian University Medical College, 80 Pradnicka St., 31-202 Kraków, Poland; 4 Department of Bioinformatics and Telemedicine, Faculty of Medicine, Jagiellonian University Medical College, Kraków, Poland; 5 Center for Digital Medicine and Robotics, Jagiellonian University Medical College, Kraków, Poland; 6 Center for Research and Innovative Technology John Paul II Hospital, Kraków, Poland

**Keywords:** Atrial fibrillation, Anticoagulation, Bleeding, Stroke, Follow-up

## Abstract

Bleeding is the most feared complication of anticoagulation. We sought to investigate whether the bleeding risk acceptance has a prognostic value during long-term follow-up in the era of direct oral anticoagulants (DOACs) among patients with atrial fibrillation (AF). We studied 167 consecutive AF outpatients [aged 68.8 SD 10.6 years; 141 (84.4%) on DOACs]. The bleeding acceptance was assessed based on the Bleeding Ratio defined as the declared maximum number of major bleedings that a patient would be willing to accept to prevent one major stroke. We recorded cerebrovascular ischemic events, major or clinically relevant non-major bleeds (CRNMB), and mortality. A median Bleeding Ratio was 4 (IQR 2–5). During follow-up of 946 patient-years, cerebrovascular ischemic events and/or death were observed in 28 patients (3.3%/ year) and major bleeding or CRNMB in 33 (4.6%/ year). The Bleeding Ratio was lower in patients who experienced cerebrovascular events or death (p = 0.004), but not bleeding. Patients with the Bleeding Ratio 0–3 were more often non-persistent to the OAC therapy, and more likely to have cerebrovascular event or die than those with higher bleeding acceptance (odds ratio 2.55; 0.95% CI 1.08–6.02) which was driven by the impact on mortality. The multiple Cox proportional hazards model showed that lower Bleeding Ratio, higher CHA_2_DS_2_-VASc score, and older age predicted cerebrovascular events or death during follow-up. AF patients who are willing to accept fewer serious bleedings to avoid major stroke during anticoagulation are more likely to experience death and/or cerebrovascular ischemic events, but not bleeding, what might be related to non-persistence.

**Supplementary Information:**

The online version contains supplementary material available at 10.1007/s11239-023-02878-8.

## Highlights

- AF patients with lower bleeding acceptance show higher risk of mortality.
- Among AF patients, low bleeding acceptance is a predictor of stroke, TIA and/or death.
- Low bleeding acceptance may lead to non-persistence to the anticoagulant regimen, therefore affect prognosis in AF.
- Bleeding acceptance should be considered when planning anticoagulation along with conventional clinical risk scores.

**Supplementary Information:**

The online version contains supplementary material available at 10.1007/s11239-023-02878-8.

## Introduction

Atrial fibrillation (AF) is a common cardiac arrhythmia affecting 0.5% of the worldwide population and major risk factor for stroke [1]. Cardioembolic strokes associated with AF are particularly severe with a 5-year recurrence rate of 21.5% and with a 5-year survival of 39%  [1–3]. Life-long oral anticoagulant therapy is a cornerstone of stroke prevention leading to a lower risk of stroke or systemic embolism by 64%, as shown for vitamin K antagonist (VKA), i.e., warfarin [4].

Life-long direct oral anticoagulants (DOAC) are preferred for stroke prevention in most AF patients [5]. The main adverse effect of OAC is bleeding. Recent network meta-analyses showed that the rate of major bleeding (MB) tends to be lower in AF patients on DOAC compared with VKA [hazard ratio (HR) 0.86; 95% confidence interval (CI) 0.74–1.01] [6]. DOAC consistently reduce the risk of intracranial bleeding by 52% and increase the risk of gastrointestinal bleeding by 25% in comparison with VKA [7]. The use of DOAC is associated with lower death incidence compared to VKA (7.76% vs. 8.42%; HR 0.92; 95% CI 0.87–0.97) [6], yet it has been suggested that VKAs with effective time in therapeutic range are at least as effective and safe as DOACs [8].

The management guidelines emphasize the importance of shared decision-making and acknowledging AF patients’ preferences [5]. Non-adherence to DOAC regimens has been reported in 29–56% of AF patients [9, 10]. It might be due to inadequate knowledge regarding their benefits and harms [11]. LaHaye et al. introduced the Bleeding Ratio as a tool to assess bleeding acceptance, which represents the maximum number of MB events patients are willing to tolerate to prevent one stroke [12]. They reported that the mean Bleeding Ratio was 4.4 among AF patients who were initiating OAC with substantial interindividual variability [12], indicating that real-life patients feared more major strokes, not bleeding. Our previous study demonstrated that the median Bleeding Ratio was 4 in AF patients and high Bleeding Ratio, defined as values more than 4, was associated with prior stroke, while low Bleeding Ratio was more commonly noted among MB survivors [13].

There are no reports on associations of long-term prognosis with MB acceptance in AF patients. We hypothesize that AF patients who are willing to accept less bleeding events while on OAC to avoid major stroke are more likely to experience major adverse events during follow-up assuming that they are less compliant, and their fears negatively impact everyday challenges of chronic anticoagulation. The aim of the study was to evaluate a prognostic value of the Bleeding Ratio declared by AF patients.

## Methods

### Patients

The study included consecutive patients with AF from an outpatient clinic at the tertiary hospital in Poland, from November 2016 to June 2019. The study population included 173 patients with documented paroxysmal, persistent, or permanent AF, were free of recent (< 3 months) cardiovascular or MB events, and taking VKA or DOAC for at least 1 month were eligible (as previously detailed [13]). Basic demographic and clinical data were collected at baseline [13]. The risk of thromboembolic events was stratified using the CHA_2_DS_2_-VASc and bleeding risk-HAS-BLED, ATRIA and ORBIT scores [14, 15]. Definitions of the comorbidities were included in Supplementary material. Appropriateness of DOAC dose adjustments was categorized according to the approved labeling for each agent [16, 17]. Non-persistence to the OAC regimen was based on self-reported instances of dose omission and/or brief interruptions.

### The Bleeding Ratio

At recruitment, we determined bleeding acceptance using the Bleeding Ratio defined as the maximum number (from 0 to 12) of MB that a given person is willing to withstand to prevent one major stroke, as previously described [13]. Following the explanation of the outcomes of stroke and MB (defined in the Supplementary material), the physician inquired about the Bleeding Ratio using a specifically designed card with consecutive numbers from 0 to 12. After selecting the number, the participants were challenged if they would be able to tolerate one more or one less MB. We used the ultimate response to determine the Bleeding Ratio.

### Follow-up

Follow-up visits were performed on a 6-month basis (a visit at the center or telephone contact with a patient or a family member) until December 2020. The clinical endpoints were: (1) cerebrovascular ischemic event and/or death; (2) bleeding [MB and/or clinically relevant non-major bleeding (CRNMB)] (defined in Supplementary material [18, 19]). Changes in OAC treatment were left to the discretion of the attending physician.

### Statistical analysis

Continuous variables were presented as means [standard deviation (SD)] or median [interquartile range (IQR)], as appropriate. The normal distribution of variables was determined using the Shapiro–Wilk test. We reported categorical variables as numbers and percentages. The chi-squared test was implemented to compare categorical variables. Differences in continuous variables between the groups were assessed using the ANOVA, Mann-Whitney U, or Kruskal–Wallis tests. The cut-off point for the Bleeding Ratio was determined based on median. The survival curve of study outcomes was analyzed according to the Kaplan–Meier method and compared by the log-rank test. The univariate and multivariate analyses of survival were conducted using the Cox proportional hazards regression models. Clinical parameters with a p-value < 0.1 were considered for potential inclusion in the multiple Cox proportional hazards model. We established the final model using stepwise regression. Thus, hazard ratios estimated from the Cox analysis were presented as relative risks with 95% CI. Statistical analyses were performed using IBM SPSS Statistics (IBM, Armonk, NY) and JMP 16.2 (SAS Institute Inc, Cary, NC). A p-value < 0.05 was considered statistically significant. The study was powered to have an 80% chance to detect hazard ratio of 0.75 at the 0.05 significance level, and 140 subjects or more were required in the study group based on estimated sample size for the Cox proportional hazards regression.

## Results

### Patient characteristics

A total of 167 patients with AF [aged 68.8 SD 10.6 years, min 39, max 94 years; 40.1% male; CHA_2_DS_2_-VASc score, median 4 (IQR 3–5); HAS-BLED − 3 (IQR 2–4)] were analyzed (Table 1). Six patients were lost to follow-up and did not differ from the remaining subjects (see Supplementary material). The majority of the patients (n = 141; 84.4%) received DOAC, including rivaroxaban (n = 70; 41.9%), apixaban (n = 37; 22.2%), and dabigatran (n = 34; 20.4%). Five patients (3.0%) received standard DOAC doses inappropriately, while for 14 patients (8.3%) DOAC doses were inappropriately reduced. The median duration of anticoagulation prior to the recruitment was 14 (IQR 7–23) months. Non-persistence was reported by 59 patients (35.3%), including short interruptions to the OAC regimen (n = 44; 26.3%) and occasionally taking lower doses (n = 20; 12.0%).

**Table 1:** Clinical outcomes during follow-up

	All	Cerebrovascular ischemic event and/or death		p-value	Major bleeding and/or CRNMB		p-value
	(n = 167)	Yes (n = 28; 16.8%)	No (n = 139; 83.2%)		Yes (n = 33; 19.8%)	No (n = 134; 80.2%)	
Age (years)	68.8 ± 10.6	74.4 ± 9.6	67.7 ± 10.6	0.001	73.1 ± 8.9	67.8 ± 10.8	0.005
Male, n (%)	67 (40.1)	9 (32.1)	58 (41.7)	0.35	14 (42.4)	53 (39.6)	0.76
Permanent AF, n (%)	44 (26.3)	10 (35.7)	34 (24.5)	0.22	12 (36.4)	32 (23.9)	0.15
Time since AF diagnosis (months)	29 (14-66)	54 (19.25–72.75)	28 (12.75–60)	0.07	39 (28.5–69)	26 (12–65.5)	0.015
Time of anticoagulant use (months)	14 (7–23)	19 (9.25–29.5)	13 (6–21)	0.12	18 (9–34.5)	13 (6.75–20.25)	0.09
CHA_2_DS_2_- VASc	4 (3–5)	5 (4.25–6)	4 (3–5)	0.002	5 (4–6)	4 (3–5)	0.034
HAS-BLED score	3 (2–4)	3 (3–4)	3 (2–4)	0.022	4 (3–4)	3 (2–3)	< 0.001
HAS-BLED score > 2, n (%)	98 (58.7)	22 (78.6)	76 (54.7)	< 0.001	28 (84.8)	70 (52.2)	< 0.001
ATRIA score	2 (1–4)	5 (1.25–6)	1 (1–4)	< 0.001	4 (1–6.5)	1 (1–4)	0.001
ORBIT score	1 (0–2)	3 (1–3)	1 (0–2)	0.001	2 (1–4)	1 (0–2)	< 0.001
Comorbidities, n (%)							
Heart failure	62 (37.1)	12 (42.9)	50 (36.0)	0.49	15 (45.5)	47 (35.1)	0.27
Arterial hypertension	142 (85.0)	25 (89.3)	117 (84.2)	0.49	29 (87.9)	113 (84.3)	0.61
Diabetes mellitus	55 (32.9)	11 (39.3)	44 (31.7)	0.43	14 (42.4)	41 (30.6)	0.20
Chronic kidney disease	47 (28.1)	11 (39.3)	36 (25.9)	0.15	15 (45.5)	32 (23.9)	0.014
Liver disease	5 (3.0)	5 (17.9)	0 (0.0)	0.31	5 (15.2)	0 (0.0)	< 0.001
Anemia	11 (6.6)	5 (17.9)	6 (4.3)	0.008	7 (21.2)	4 (3.0)	< 0.001
Prior cerebrovascular ischemic event	59 (35.3)	11 (39.3)	48 (34.5)	0.63	12 (36.4)	47 (35.1)	0.89
Vascular disease	58 (34.7)	13 (46.4)	45 (32.4)	0.15	15 (45.5)	43 (32.1)	0.15
Past major bleeding	33 (19.8)	9 (32.1)	24 (17.3)	0.07	9 (27.3)	24 (17.9)	0.23
Medication, n (%)				0.06			0.007
DOAC	141 (84.4)	24 (85.7)	117 (84.2)		24 (72.7)	117 (87.3)	
VKA	12 (7.2)	3 (10.7)	9 (6.5)		7 (21.2)	5 (3.7)	
LMWH*	6 (3.6)	0 (0.0)	6 (4.3)		1 (3.0)	5 (3.7)	
No anticoagulation	8 (4.8)	1 (3.6)	7 (5.0)		1 (3.0)	7 (5.2)	
Concomitant antiplatelet therapy	47 (28.1)	9 (32.1)	38 (27.3)	0.61	13 (39.4)	34 (25.4)	0.11
Change in OAC	40 (24.0)	5 (17.9)	35 (25.2)	0.41	17 (51.5)	23 (17.2)	< 0.001
VKA previously	73 (43.7)	17 (60.7)	56 (40.3)	0.047	18 (54.5)	55 (41.0)	0.16
Non-persistence**	59 (35.3)	18 (64.3)	41 (29.5)	< 0.001	12 (36.4)	47 (35.1)	0.89
Short interruptions	44 (26.3)	12 (42.9)	32 (23.0)	0.03	8 (24.2)	36 (26.9)	0.76
Omission of doses	20 (12.0)	8 (28.6)	12 (8.6)	0.003	4 (12.1)	16 (11.9)	0.98
Inappropriate DOAC dose reduction	14 (8.4)	2 (7.1)	12 (8.6)	0.80	3 (9.1)	11 (8.2)	0.87
The Bleeding Ratio	4 (2–5)	2 (1.25–4)	3 (2–4)	0.004	3 (2–4)	4 (2–5)	0.24

Data reported as number (percentage), mean (standard deviation), or median (interquartile range).

*AF* atrial fibrillation, *CHA*_2_*DS*_2_-*VASc* Congestive Heart Failure, Hypertension, Age 65–74/Age ≥ 75, Diabetes Mellitus, Prior Stroke or Transient Ischemic Attack, Vascular Disease, Female, *LMWH* low-molecular-weight heparin, *CRNMB* clinically relevant non-major bleeding, *DOAC* direct oral anticoagulant, *OAC* oral anticoagulant, *VKA* vitamin K antagonist

*Largely due to planned invasive procedures within few days

**None of the patients declared permanent OAC withdrawal

**Fig. 1:**
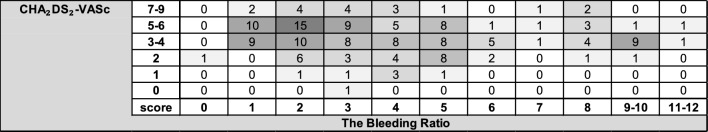
Distribution of patients by the Bleeding Ratio and the CHA_2_DS_2_-VASc score

The median Bleeding Ratio was 4 (IQR 2–5). Eighty-two patients (49.1%) with the Bleeding Ratio less than 4 (Fig. 1) represented the low Bleeding Ratio group, whereas the remainder represented the high Bleeding Ratio group (n = 85; 50.9%; Supplementary Table S1). The low Bleeding Ratio was associated with higher HAS-BLED score (Supplementary Table S1).

### Follow-up

The median duration of follow-up was 51 (IQR 45–67) months (946 patient-years). A change of OAC was recorded in 40 cases (24%), mainly the switch to apixaban at reduced (n = 13; 32.5%) or full (n = 24; 60.0%) doses, primarily due to renal impairment. At last contact as few as 6 patients (3.6%) were on VKA.

**Fig. 2:**
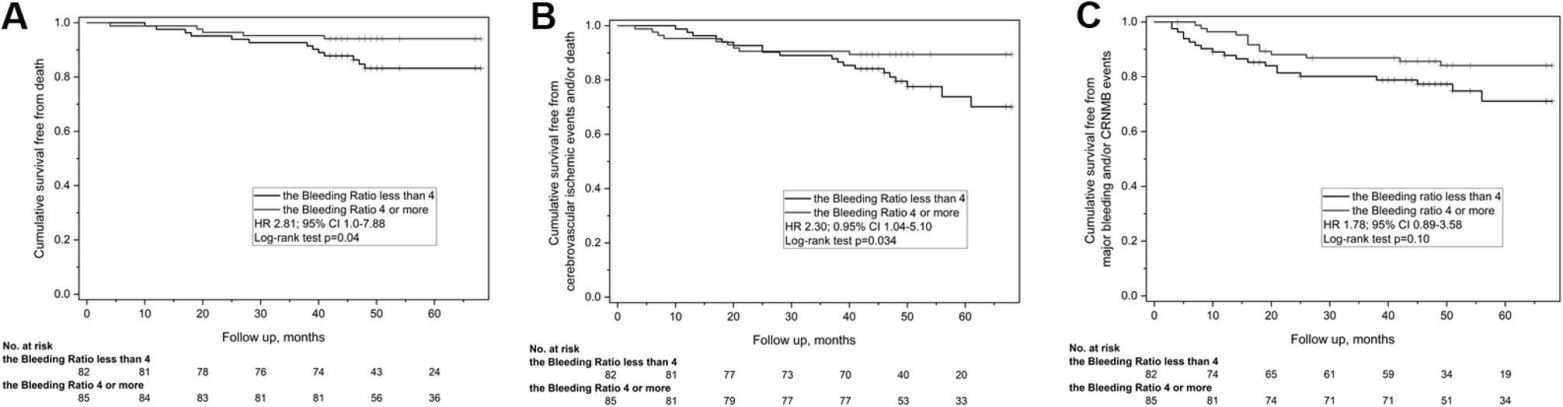
Kaplan–Meier curves in patients with atrial fibrillation for **a** death; **b** the cerebrovascular ischemic event and/or death; **c** major bleedings and/or clinically relevant non-major bleeding events. *CI* confidence interval, *CRNMB* clinically relevant non-major bleeding, *HR* hazard ratio

Eighteen (2.5% per year) patients died during follow-up. Cardiovascular deaths related to coronary artery disease prevailed. Mortality was associated with older age (p < 0.001), higher CHA_2_DS_2_-VASc score (p < 0.001), higher HAS-BLED score (p = 0.002), the occurrence of chronic kidney disease (p = 0.029), vascular disease (p = 0.013), permanent AF (p = 0.003), longer time since AF diagnosis (p = 002) and initiation of anticoagulant (p = 0.031). Notably, low Bleeding Ratio in the range of 0–3 was associated with increased mortality. Patients in the low Bleeding Ratio group were three times more likely to experience death during follow-up as compared to the remainder [15.9% vs. 5.9%; odds ratio (OR) 3.01; 0.95% CI 1.02–8.88]. Kaplan–Meier curves confirmed increased mortality in the former group (HR 2.81; 95% CI 1.0–7.88; log-rank test p = 0.04; Fig. 2a).

**Fig. 3:**
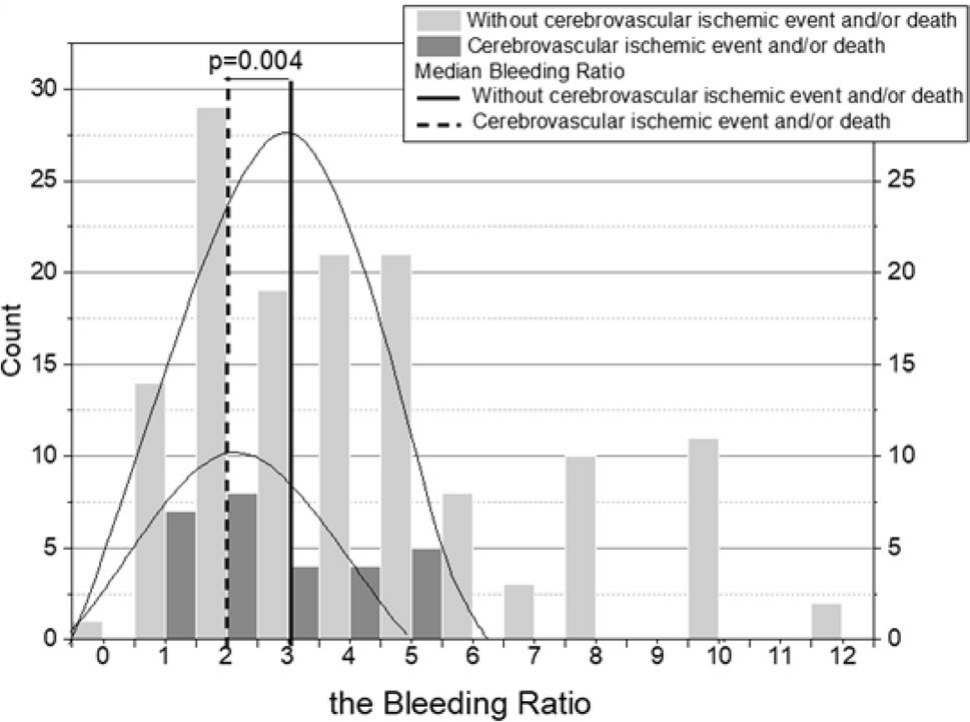
Distribution of the Bleeding Ratio in patients with atrial fibrillation depending on the occurrence of the cerebrovascular ischemic event and/or death

The compound outcome (cerebrovascular ischemic event and/or death) was observed in 28 patients (3.3% per year), being associated with older age, prior use of VKA, higher CHA_2_DS_2_-VASc and HAS-BLED scores (Table 1). The baseline Bleeding Ratio was lower in patients who experienced the composite endpoint [median, 2 (IQR 1.25–4) vs. 3 (IQR 2–4); Fig. 3], and this effect was mainly driven by mortality. The low Bleeding Ratio group had higher risk of this outcome (HR 2.30; 0.95% CI 1.04–5.10; log-rank p = 0.034; Fig. 2b). There was a more than twice greater odds of occurrence of cerebrovascular ischemic event or death in the low Bleeding Ratio group (23.2% vs. 10.5%; OR 2.55; 0.95% CI 1.08–6.02). The multiple Cox proportional hazards model showed that low Bleeding Ratio, along with higher CHA_2_DS_2_-VASc score and age, were predictors of the cerebrovascular ischemic event and/or death during follow-up (Table 2).

**Table 2:** The multiple Cox proportional hazards model for predictors of the cerebrovascular ischemic event and/or death in AF patients

Variable	Univariate analysis		Multivariate analysis*	
	Risk ratio (0.95% CI)	p-value	Risk ratio (0.95% CI)	p-value
The Bleeding ratio	0.75 (0.62–0.92)	0.006	0.79 (0.62–0.99)	0.041
CHA_2_DS_2_- VASc score	1.39 (1.12–1.71)	0.002	1.30 (1.00–1.68)	0.051
Age per 10 years	1.13 (1.04–1.21)	0.003	1.02 (1.04–1.13)	0.071
VKA previously	2.05 (0.96–4.38)	0.063		

*CI* confidence interval, other—see Table 1

*Adjusted for age, sex, heart failure, hypertension and diabetes

Thirty-three patients had bleeding events (4.6% per year), including 15 MB (2.1% per year), which were associated with older age, chronic kidney disease, longer time since AF diagnosis, and the change of OAC use during follow-up (Table 1). The multiple Cox proportional hazards model indicated that only older age (per 10 years) predicts the bleeding risk during follow-up (adjusted HR 1.10; 0.95% CI 1.03–1.18; p = 0.006). Low Bleeding Ratio was not associated with the risk of MB or CRNMB, analyzed separately or combined, during follow-up with a slight insignificant trend toward higher risk as shown in Fig. 2c (HR 1.78; 95% CI 0.89–3.58; log-rank p = 0.10).

Patients on inappropriately reduced DOAC doses did not differ from the rest of the study group in terms of the Bleeding Ratio (p = 0.14) or occurrence of clinical outcomes (cerebrovascular ischemic event and/or death, p = 0.80; bleeding, p = 0.87). Non-persistent patients exhibited a lower Bleeding Ratio compared to those who adhered to the regimen [median 2 (IQR 2–3) vs. 5 (IQR 3–6); p < 0.001]. Death and cerebrovascular ischemic events (but not bleedings) were more commonly observed among patients reporting non-persistence than the rest of the study group (64.3% vs. 29.5%; p < 0.001).

## Discussion

Our study is the first to demonstrate that self-declared low bleeding acceptance, defined as less than 4 MB the patient would be ready to endure to prevent one major stroke, is related to an increased risk of cerebrovascular ischemic events and death in anticoagulated AF patients during long-term follow-up. This finding indicates that a straightforward, user-friendly tool—the Bleeding Ratio—can possess predictive value, particularly concerning mortality, largely of cardiovascular origin, beyond established risk factors as advanced age, vascular disease, and an increased CHA_2_DS_2_-VASc score. Contrary to patients’ concerns, the willingness to accept up to 12 MB to prevent one major stroke had no influence on MB or CRNMB. Our study provides additional evidence that AF patients’ attitude towards their perceived risk of MB versus stroke holds clinical significance, suggesting that modifying bleeding acceptance could impact long-term prognosis. Considering that bleeding risk acceptance can be partially altered through targeted education, it can be speculated that enhancing bleeding acceptance by increasing knowledge of AF and anticoagulant therapy may have a substantial effect on the prognosis, likely due to improved adherence and compliance.

We employed a simple and practical approach to determine bleeding acceptance in a real-life setting introduced in 2014 [12] with minor modifications—we considered death as a potential outcome of both major stroke and MB [13]. This parameter is useful in identifying risk-aware and risk-averse users of antithrombotic treatment. It allows individual patients to express their opinions and assists in guiding anticoagulation decisions. The average Bleeding Ratio in our study was nearly identical to that reported by LaHaye et al. [12], with 4 bleedings accepted to prevent one severe stroke. We provided additional evidence that contemporary AF patients on OAC, mainly on DOAC, perceive major stroke as more dangerous than MB.

This emphasizes the importance of considering patients’ preferences, not solely relying on clinical scores, when choosing appropriate antithrombotic management [20]. The broadly used CHA_2_DS_2_-VASc score, a recognized instrument for risk stratification in AF, has an impact on prognosis, which aligns with our current findings. However, concerns have been raised regarding the limitations of the CHA_2_DS_2_-VASc score, including inadequate specificity in predicting AF-related versus non-AF-related ischemic events and misidentification of low-risk patients [21, 22]. A recent study showed that only 47% of AF patients were prescribed OACs by primary care doctors, regardless of their CHA_2_DS_2_-VASc score [23]. Additionally, patients appear to be less sensitive to bleeding risk than doctors [24]. Assessing bleeding acceptance alongside thromboembolic risk evaluation allows the coupling of estimated clinical risk with patients’ perspectives, whether they are risk-aware or risk-averse [25]. Our observation highlights the potential role of the Bleeding Ratio assessment in AF patients, suggesting that its implementation is worthwhile. It can be postulated that the CHA_2_DS_2_-VASc score and Bleeding ratio used together may better predict AF-related thromboembolic events, mortality and enable targeted educational efforts for the risk-averse subgroups.

Notably, we observed less commonly AF patients who were unwilling to accept *any* bleedings to prevent major stroke, as compared to the Canadian study [12]. This might be attributed to the wider usage of generally safer and more user-friendly DOAC. Even if the Bleeding Ratio assessment might be limited by cognitive ability to comprehend the concept of balance between safety and effectiveness of the OAC therapy, our study demonstrated that real-life AF patients are capable of making decisions and expressing their bleeding risk perception, which holds prognostic significance and should not be disregarded.

Predictably, older patients exhibited lower Bleeding Ratio, likely due to higher prevalence of comorbidities and higher overall risk of adverse events. This relation between the baseline Bleeding Ratio and age has consistently been observed in previous research [12, 13, 26]. Age is one of the factors influencing bleeding acceptance, so the impact of the Bleeding Ratio is driven by other valid confounders and affects prognosis in terms of the occurrence of cerebrovascular events or mortality.

The present study yielded adverse event rates that are comparable to those reported in other cohort studies involving anticoagulated AF patients, with the stroke incidence of 1.5–2.5% per year and the incidence of MB of 2–4% per year [7]. This indicates that our study population is representative of contemporary European AF populations. Our study showed lower mortality rate mortality rate compared to the CRAFT study [27] (6%/ year) or similar compared to the ROCKET-AF study [28] (1.9%/ year for rivaroxaban; 2.2/year for warfarin), and patients’ concerns about MB may contribute to these figures.

Importantly, we observed the association between low acceptance of bleeding and an increased risk of thromboembolic events when analyzed together with mortality. It should be highlighted that this effect was mainly driven by higher mortality largely caused of coronary ischemia. This observation implies that AF patients with low Bleeding Ratio should receive close surveillance and educational efforts to improve bleeding acceptance. As recommended [5], personalized information regarding benefits and risks of OACs and regular assessment of individual stroke risk should be provided to enhance compliance and adherence, particularly in AF patients at an elevated risk of bleeding (elderly, with comorbidities) [29]. Previous research have demonstrated an association between the educational attainment of AF patients and the occurrence of ischemic stroke and mortality [30]. Nevertheless, it remains to be determined whether educating about benefits and risks of OACs can increase bleeding acceptance and subsequently reduce the risk of adverse clinical outcomes in AF patients.

Bleedings, the most concerning adverse events among OAC users, were the predominant outcome in our study (4.6% annually) with the greatest impact of advanced age, as expected. Prior MB was a crucial predictor of low Bleeding Ratio in AF patients and acute stroke survivors [26]. Nevertheless, we did not observe any association between bleeding acceptance and the incidence of major or CRNMB during follow-up. It indicates good adherence related to low-level of fears in this regard. Prior MB or predisposition to bleeding during OAC use might contribute to reduced compliance to medications among AF patients, resulting in a gradual decrease of persistence in OAC use [31]. In our study, a subset of participants exhibited suboptimal adherence to OAC therapy related to low Bleeding Ratio, due to short OAC interruptions or skipping doses, which is in line with the results of Ozaki et al., demonstrating that approximately one-third of patients may not adhere to the DOAC regimen, leading to unfavorable clinical outcomes [9]. Importantly, our study demonstrated that non-persistence to the OAC regimen is associated with lower bleeding acceptance, potentially contributing to the increased occurrence of death or cerebrovascular ischemic events among non-persistent patients. Notably, among patients receiving reduced doses of DOACs without clear indications (referred to as a suboptimal regimen), their tolerance towards potential bleeding was similar to that of the remaining patients and it did not affect the occurrence of clinical outcomes.

### Study limitations

Firstly, the sample size was relatively small, although this single-center study was adequately powered to demonstrate the impact of bleeding acceptance on long-term prognosis. Secondly, we did not evaluate potential socio-economic confounders, mental health status, and education that may have influenced the findings. In addition, the assessment of persistence to OAC therapy was evaluated based on self-declaration. Finally, the Bleeding Ratio was assessed only once at enrollment, assuming this factor is not subject to change over time, especially since no systemic intervention had been implemented to modify bleeding acceptance. It remains to be established whether the Bleeding Ratio can change over time.

## Conclusions

We demonstrated for the first time that low bleeding acceptance might be a prognostic factor for the incidence of all-cause mortality and cerebrovascular ischemic events in AF patients on OACs, whereas this observation is mainly driven by mortality rates. Our observations warrant further studies to validate the results in other populations of AF patients. In our opinion, low bleeding acceptance, regardless of the scoring system used, should be taken into consideration and increase clinical surveillance as it might lead to non-persistence. By implementing the evaluation of bleeding acceptance (e.g., using the Bleeding Ratio) physicians may engage patients into shared-decision making in clinical practice and acknowledge their values and preferences.

### Supplementary Information

Below is the link to the electronic supplementary material.

Supplementary material 1 (DOCX 34.6 kb)
