# Supplementary material for: Low bleeding acceptance is associated with increased death risk in patients with atrial fibrillation on oral anticoagulation
Source: J Thromb Thrombolysis. 2023 Aug 19;57(1):155–63. doi: 10.1007/s11239-023-02878-8 (PMC10830776; doi:10.1007/s11239-023-02878-8)
Supplement: Supplementary file 1 — Supplementary material 1 (DOCX 34.6 kb) [file 11239_2023_2878_MOESM1_ESM.docx]

**Supplementary material**

**Low Bleeding Acceptance Is Associated With Increased Death Risk In Patients With Atrial Fibrillation On Oral Anticoagulation**

Rusin Gabriela MD^a^, Konieczyńska Małgorzata MD PhD^b,c^, Natorska Joanna PhD ^c^, Malinowski Krzysztof Piotr^d,e^, Undas Anetta MD PhD^c,f^ *

^a^ Department of Neurology, University Hospital, Krakow, Poland

^b^ Department of Diagnostic Medicine, John Paul II Hospital, Kraków, Poland

^c^ Department of Thromboembolic Disorders, Institute of Cardiology, Jagiellonian University Medical College, Kraków, Poland

^d^ Department of Bioinformatics and Telemedicine, Faculty of Medicine, Jagiellonian University Medical College, Kraków, Poland

^e^ Center for Digital Medicine and Robotics, Jagiellonian University Medical College, Kraków, Poland

^f^ Center for Research and Innovative Technology John Paul II Hospital, Kraków, Poland

* Corresponding author: Jagiellonian University Medical College, 80 Pradnicka St., 31-202 Krakow, Poland. Tel.: +48 12 6143004; fax: +48 12 4233900. E-mail address: [mmundas@cyf-kr.edu.pl](mailto:mmundas@cyf-kr.edu.pl)

**Supplementary Table S1. The low and high Bleeding Ratio groups**

|  | All (n=167) | The Bleeding ratio <4  (n=82; 49.1%) | The Bleeding ratio ≥4  (n=85; 50.9%) | p-value |
| --- | --- | --- | --- | --- |
| Age [years] | 68.8 ± 10.6 | 73.5 ± 9.5 | 64.3 ± 9.8 | <0.001 |
| Male, n (%) | 67 (40.1) | 31 (37.8) | 36 (42.4) | 0.55 |
| Permanent AF, n (%) | 44 (26.3) | 32 (39.0) | 12 (14.1) | <0.001 |
| Time since AF diagnosis [months] | 29 (14-66) | 42 (19.5-84) | 19 (11.5-50.5) | <0.001 |
| Time of anticoagulant use [months] | 14 (7-23) | 13 (6-23) | 14 (7-23.75) | 0.65 |
| CHA_2_DS_2_-VASc score | 4 (3-5) | 5 (3-6) | 4 (3-5) | 0.07 |
| HAS-BLED score | 3 (2-4) | 3 (2-4) | 2 (2-3) | <0.001 |
| HAS-BLED score >2, n (%) | 98 (58.7) | 61 (74.4) | 37 (43.5) | <0.001 |
| ORBIT score | 1 (1-3) | 2 (1-3) | 1 (0-1) | <0.001 |
| ATRIA score | 2 (1-3) | 2 (2-3) | 1 (1-2) | <0.001 |
| Comorbidities, n (%) |  |  |  |  |
| Heart failure | 62 (37.1) | 40 (48.8) | 22 (25.9) | 0.002 |
| Arterial hypertension | 142 (85.0) | 66 (80.5) | 76 (89.4) | 0.11 |
| Diabetes mellitus | 55 (32.9) | 27 (32.9) | 28 (32.9) | 0.99 |
| Chronic kidney disease | 47 (28.1) | 19 (23.2) | 28 (32.9) | 0.09 |
| Liver disease | 5 (3.0) | 1 (1.2) | 4 (4.7) | 0.19 |
| Anemia | 11 (6.6) | 8 (9.8) | 3 (3.5) | 0.11 |
| Prior cerebrovascular ischemic event | 59 (35.3) | 21 (25.6) | 38 (44.7) | 0.01 |
| Vascular disease | 58 (34.7) | 30 (36.6) | 28 (32.9) | 0.62 |
| Past major bleeding | 33 (19.8) | 27 (32.9) | 6 (7.1) | <0.001 |
| Medication, n (%) |  |  |  | 0.13 |
| DOAC | 141 (84.4) | 65 (79.3) | 76 (89.4) |  |
| VKA | 12 (7.2) | 7 (8.5) | 5 (9.4) |  |
| LMWH | 6 (3.6) | 4 (4.9) | 2 (2.4) |  |
| No anticoagulation | 8 (4.8) | 6 (7.3) | 2 (2.4) |  |
| Concomitant antiplatelet therapy | 47 (28.1) | 25 (30.5) | 22 (25.9) | 0.51 |
| Change in OAC | 35 (21.0) | 21 (25.6) | 14 (16.5) | 0.15 |
| Non-persistence | 59 (35.3) | 49 (59.8) | 10 (11.8) | <0.001 |
| Short interruptions | 44 (26.3) | 39 (47.6) | 5 (5.9) | <0.001 |
| Omission of doses | 20 (12.0) | 14 (17.1) | 6 (7.1) | 0.046 |
| Inappropriate DOAC dose reduction | 14 (8.4) | 8 (9.8) | 6 (7.1) | 0.53 |
| Follow-up, n (%) |  |  |  |  |
| Death | 18 (10.8) | 13 (15.9) | 5 (5.9) | 0.038 |
| Death/cardiovascular ischemic event | 28 (16.8) | 19 (23.2) | 9 (10.6) | 0.030 |
| Bleeding | 33 (19.6) | 20 (24.4) | 13 (15.3) | 0.14 |

Data reported as number (percentage), mean (standard deviation) or median (interquartile range) Abbreviations: AF, atrial fibrillation; CHA_2_DS_2_-VASc, Congestive Heart Failure, Hypertension, Age 65-74/ Age ≥75, Diabetes Mellitus, Prior Stroke or Transient Ischemic Attack, Vascular Disease, Female; LMWH; low-molecular-weight heparin; DOAC, direct oral anticoagulant; OAC, oral anticoagulant; VKA, vitamin K antagonist

**The determination of the Bleeding Ratio**

The physician provided standardised information to each patient regarding major stroke and major bleeding.  In order to explain to the participants what major stroke and its outcomes are, we used the description as follows:

- A stroke is a medical emergency which happens when the blood is not sufficiently supplies to the part of your brain. The brain cells do not get enough oxygen or nutrients, and begin to die. This process is irreversible.
- Your extremities will be very weak, hard to move or totally paralyzed.
- Your speech can be slurred, hard to understand. You might not be able to communicate with others.
- You might will have to spend all of your time either in a bed or in a wheelchair.
- You will not be able to run errands, do your chores, as you will be dependable on the constant help from other people.
- You will need someone to take care of you or go to a nursing home.
- You might die.

We explained to the patients what a major bleeding is by providing the following information:

- A major bleeding is a medical emergency which involves significant loss of blood from your digestive tract over a short period of time.
- For a couple of days, you will feel sick and then you lose lots of blood by vomiting abruptly.
- You will have to spend at least several days in a hospital.
- A blood transfusion is likely to be needed.
- The doctors will need to put a tube down your food pipe to make clear what caused the bleeding.
- By the time you recover, you will feel fatigue for at least a month.
- You might die.

**Definitions**

Ischemic stroke was defined as an acute episode of focal neurological deficit of vascular etiology based on clinical or imaging evidence. Transient ischemic attack (TIA) was defined as a transient (<24 hours) episode of neurological dysfunction of vascular origin without imaging abnormalities. [1] Cerebrovascular ischemic events were diagnosed based on World Health Organization criteria using the definitions of stroke and transient ischemic attack.

Major bleeding was defined according to the International Society on Thrombosis and Haemostasis as symptomatic bleeding in a critical area or organ, or bleeding causing a fall in the haemoglobin level of at least 20 g/L, or leading to transfusion of 2 or more units of whole blood or red cells. [2,3]

Clinically relevant non-major bleeding was defined as clinically overt bleeding that led to a hospital stay, required medical intervention, and did not satisfy the criteria for major bleeding. [4]

Arterial hypertension was diagnosed when systolic or diastolic blood pressure ≥ 140 mm Hg and/or ≥ 90 mm Hg, respectively, and/or during antihypertensive treatment.

Diabetes was diagnosed according to the World Health Organization criteria [5].

Heart failure was diagnosed based on the European Society of Cardiology guidelines for acute and chronic heart failure [6].

Specific types of AF (persistent and permanent) were defined according to European Society of Cardiology [7].

Vascular disease was diagnosed as a history of peripheral arterial disease, coronary arterial disease or myocardial infarction.

Anemia in men was defined as hemoglobin <13 g/dL or hematocrit <40%, whereas for women - hemoglobin <12 g/dL or hematocrit <36%.

Chronic kidney disease was defined as estimated glomerular filtration rate lower than 60 ml/min/1.73 m^2^ for at least 3 months.

Liver disease was defined as known liver cirrhosis or alanine or aspartate aminotransferases above 3 fold the upper limit of the reference range and bilirubin 2 fold the upper limit of the reference range.

The clinical risk of thromboembolic events was stratified using the CHA_2_DS_2_-VASc score. [8]

The clinical risk of bleeding events was assessed using the HAS-BLED, ATRIA and ORBIT scores. [9-11]

**Baseline characteristics of the patients who were lost to follow-up**

n=6 (male: n=2)

Mean age 65.7±10.9

Median of the Bleeding Ratio 5 (IQR 2-5.25)

Anticoagulants: dabigatran (n=3); apixaban (n=1); rivaroxaban (n=2)

Comorbidities: Heart failure (n=2); hypertension (n=6); diabetes (n=2)

**References:**

[1] Sacco RL, Kasner SE, Broderick JP, et al. An updated definition of stroke for the 21st century: a statement for healthcare professionals from the American Heart Association/American Stroke Association [published correction appears in Stroke. 2019 Aug;50(8):e239]. Stroke. 2013;44(7):2064-2089

[2] Undas A, Drabik L, Potpara T. Bleeding in anticoagulated patients with atrial fibrillation: practical considerations. Kardiol Pol. 2020;78(2):105-116

[3] Schulman S, Angerås U, Bergqvist D, et al. Definition of major bleeding in clinical investigations of antihemostatic medicinal products in surgical patients. J Thromb Haemost. 2010;8(1):202-204

[4] Kaatz S, Ahmad D, Spyropoulos AC, Schulman S; Subcommittee on Control of Anticoagulation. Definition of clinically relevant non-major bleeding in studies of anticoagulants in atrial fibrillation and venous thromboembolic disease in non-surgical patients: communication from the SSC of the ISTH. J Thromb Haemost. 2015;13(11):2119-2126

[5] Gorczyca I, Michta K, Pietrzyk E, et al. Predictors of post-operative atrial fibrillation in patients undergoing isolated coronary artery bypass grafting. Kardiol Pol. 2018; 76(1): 195-201, doi: 10.5603/KP.a2017.0203.

[6] McDonagh TA, Metra M, Adamo M, et al. 2021 ESC Guidelines for the diagnosis and treatment of acute and chronic heart failure. Eur Heart J. 2021; 42(36): 3599-3726, doi: 10.1093/eurheartj/ehab368.

[7] Hindricks G, Potpara T, Dagres N, et al. 2020 ESC Guidelines for the diagnosis and management of atrial fibrillation developed in collaboration with the European Association for Cardio-Thoracic Surgery (EACTS): The Task Force for the diagnosis and management of atrial fibrillation of the European Society of Cardiology (ESC) Developed with the special contribution of the European Heart Rhythm Association (EHRA) of the ESC. Eur Heart J. 2021; 42(5): 373-498, doi: 10.1093/eurheartj/ehaa612.

[8] Lip GY, Nieuwlaat R, Pisters R, Lane DA, Crijns HJ. Refining clinical risk stratification for predicting stroke and thromboembolism in atrial fibrillation using a novel risk factor-based approach: the euro heart survey on atrial fibrillation. Chest. 2010 Feb;137(2):263-72

[9] Pisters R, Lane DA, Nieuwlaat R, de Vos CB, Crijns HJ, Lip GY. A novel user-friendly score (HAS-BLED) to assess 1-year risk of major bleeding in patients with atrial fibrillation: the Euro Heart Survey. Chest. 2010;138(5):1093-1100. doi:10.1378/chest.10-0134

[10] O’Brien EC, Simon DN, Thomas LE, et al. The ORBIT bleeding score: a simple bedside score to assess bleeding risk in atrial fibrillation. Eur Heart J. 2015;36(46):3258-3264. Doi:10.1093/eurheartj/ehv476

[11] Fang MC, Go AS, Chang Y, et al. A new risk scheme to predict warfarin-associated hemorrhage: The ATRIA (Anticoagulation and Risk Factors in Atrial Fibrillation) Study. J Am Coll Cardiol. 2011;58(4):395-401. doi:10.1016/j.jacc.2011.03.031
